# Auditory salience using natural soundscapes

**DOI:** 10.1121/1.4979055

**Published:** 2017-03-28

**Authors:** Nicholas Huang, Mounya Elhilali

**Affiliations:** Department of Biomedical Engineering, The Johns Hopkins University, Baltimore, Maryland 21218, USA; Department of Electrical and Computer Engineering, Center for Language and Speech Processing, The Johns Hopkins University, Baltimore, Maryland 21218, USA

## Abstract

Salience describes the phenomenon by which an object stands out from a scene. While its underlying processes are extensively studied in vision, mechanisms of auditory salience remain largely unknown. Previous studies have used well-controlled auditory scenes to shed light on some of the acoustic attributes that drive the salience of sound events. Unfortunately, the use of constrained stimuli in addition to a lack of well-established benchmarks of salience judgments hampers the development of comprehensive theories of sensory-driven auditory attention. The present study explores auditory salience in a set of dynamic natural scenes. A behavioral measure of salience is collected by having human volunteers listen to two concurrent scenes and indicate continuously which one attracts their attention. By using natural scenes, the study takes a data-driven rather than experimenter-driven approach to exploring the parameters of auditory salience. The findings indicate that the space of auditory salience is multidimensional (spanning loudness, pitch, spectral shape, as well as other acoustic attributes), nonlinear and highly context-dependent. Importantly, the results indicate that contextual information about the entire scene over both short and long scales needs to be considered in order to properly account for perceptual judgments of salience.

## I. INTRODUCTION

Attention is at the center of any study of sensory information processing in the brain.^1^ It describes mechanisms by which the brain focuses both sensory and cognitive resources on important elements in the stimulus.^2^ Intuitively, the brain has to sort through the flood of sensory information impinging on its senses at every instance and put the spotlight on a fraction of this information that is relevant to a behavioral goal. For instance, carrying a conversation in a crowded restaurant requires focusing on a specific voice to isolate it from a sea of competing voices, noises, and other background sounds. It is common in the literature to employ the term attention to refer to “voluntary attention” or “top-down attention” that is directed to a target of interest.^3^

A complementary aspect to these processes is bottom-up attention, also called salience or saliency, which describes qualities of the stimulus that *attract* our attention and make certain elements in the scene stand out relative to others.^4^ This form of attention is referred to as bottom-up since it is stimulus-driven and fully determined by attributes of the stimulus and its context. In many respects, salience is involuntary and not dictated by behavioral goals. A fire alarm will attract our attention regardless of whether we wish to ignore it or not. While salience is compulsory, it is modulated by top-down attention. As such, the study of salience *alone* away from influences of top-down attention is a challenging endeavor.

Much of what we know today about salience in terms of neural correlates, perceptual qualities, and theoretical principles comes from vision studies.^5,6^ When an object has a different color, size, or orientation relative to neighboring objects, it stands out more, making it easier to detect. Behavioral paradigms such as visual search or eye tracking tasks are commonly used to investigate the exact perceptual underpinnings of visual salience.^7–10^ This rich body of work has resulted in numerous resources such as standard databases which enable researchers to probe progress in the field by working collectively to replicate human behavior in response to different datasets such as still natural images, faces, animate vs inanimate objects, videos, etc.^11^ Using common references not only contributes to our understanding of salience in the brain, but has tremendous impact on computer vision applications ranging from robotics to medical imaging and surveillance systems.^12,13^

In contrast, the study of salience in audition remains in its infancy (see Ref. 14). A number of issues are hampering progress in studies of auditory salience. First, there is a lack of agreed upon behavioral paradigms that truly probe the involuntary, stimulus-driven nature of salience. Most published studies used a detection paradigm where a salient object is embedded in a background scene, and subjects are asked to detect whether a salient event is present or not; or to compare two scenes and detect or match salience levels across the two scenes.^15–18^ In these setups, salient events are determined by an experimenter based on a clear distinction from the background scene. As such, their timing provides a ground truth against which accompanying models are tested, and the relevance of various features is assessed. While quite valuable, this approach confines the analysis of salient features and sound events to predetermined hypotheses assumed in the study. A complementary approach favored in other studies used annotation where listeners are asked to mark timing of salient events in a continuous recording.^19^ This paradigm puts less emphasis on a choice of events determined by an experimenter, and adopts a more stimulus-driven approach that polls responses across subjects to determine salience. Still, this approach presents its own limitation in terms of controlling top-down and cognitive factors since listeners are presented with a single scene whose context might bias their judgment of stimulus-driven salience. A different direction favored in other bodies of work focuses on the “distraction” effect induced by auditory salience of sound events or patterns.^20,21^ This approach takes into account effects of attentional load through the use of competing tasks, but it has yet to provide a broad canvassing of the acoustic features that render certain sound events more salient than others.

A related challenge to the study of auditory salience is a lack of standard datasets and stimulus baselines that guide the development of theoretical frameworks and explain why certain sound events stand out in a soundscape. Of the few studies conducted on auditory salience, most have used well-controlled stimuli, like simple tones^22^ or short natural recordings in controlled uniform backgrounds.^15,17,18^ A few studies incorporated more rich natural scenes using recordings from the BU Radio News Corpus^23^ and the AMI Corpus.^19^ Still, both of the latter databases consist of scenes with relatively homogeneous compositions, both in terms of audio class (mostly speech interspersed with other sounds) and density (mostly sparse with rare instances of overlapping sound objects). The lack of a diverse and realistic selection of natural soundscapes in the literature results in a narrow view of what drives salience in audition. In turn, theoretical frameworks developed to model empirical data remain limited in scope and fail to generalize beyond the contexts for which they were developed.^14^

The current study aims to break this barrier and offer a baseline database for auditory salience from an unconstrained choice of stimuli. This collection includes a variety of complex natural scenes, which were selected with a goal of representing as many types of real world scenarios as possible. The database was not developed as underlying paradigm to accompany a specific model. Rather, it was designed to challenge existing models of auditory salience and stimulate investigations into improved theories of auditory salience. The sound dataset and its accompanying behavioral measures are intended for public use by the research community and can be obtained by contacting the corresponding author.

The experimental paradigm collected behavioral responses from human volunteers while listening to two competing natural scenes, one in each ear. Subjects indicated continuously which scene attracted their attention. This paradigm was chosen to address some of the limitations of existing approaches in studies of auditory salience. Salience judgments are determined by the scenes themselves with no prearranged placement of specific events or salience ground truth. Moreover, employing two competing scenes in a dichotic setting disperses effects of top-down attention and allows salient events to attract listeners' attention in a stimulus-driven fashion; hence expanding on the previously used annotation paradigm used by Kim *et al.*^19^ Still, studying salience using these sustained natural scenes comes with many limitations and challenges. First, it is impossible to fully nullify the effects of voluntary attention in this paradigm. Subjects are actively and continuously indicating scenes that attract their attention. To mitigate this issue, the use of a large number of listeners and assessment of behavioral consistency across responses averages out voluntary attentional effects, and yields consistent response patterns reflecting signal-driven cues. Second, certain sounds may induce preferential processing relative to others. The use of real world recordings inherently provides context that may or may not be familiar to some listeners. That is certainly the case for speech sounds, certain melodies (for subjects who are musicians or music-lovers) as well as other familiar sounds. While this is an aspect that again complicates the study of auditory salience for subsets of listeners, the use of a large pool of volunteers will highlight the behavior of average listeners, leaving the focus on specific sounds of interest as a follow-up analysis. In addition, it is the complex interactions in a realistic setting that limits the translation of salience models developed in the laboratory to real applications. Third, two competing auditory scenes are presented simultaneously, to further divide the subjects' voluntary attention. As such, the study presents an analysis of salience “relative” to other contexts. By counter-balancing relative contexts across listeners, we are again probing average salience of a given sound event or scene regardless of context. Still, it is worth noting that the use of dichotic listening may not be very natural but does simulate the presence of competing demands on our attentional system in challenging situations. Finally, subjects are continuously reporting which scene attracts their attention; rather than providing behavioral responses after the stimulus. By engaging listeners throughout the scene presentation, the paradigm aims to minimize effects of different cognitive factors including voluntary attention and memory that can cloud their report. In the same vein, this study focuses on the onset of shifts in attention, before top-down attention has time to “catch up.” Throughout the paradigm, pupil dilation is also acquired, hence providing a complementary account of salience that is less influenced by top-down attentional effects.

In this report, we provide an overview of the dataset chosen as well as analyses of the bottom-up attention markers associated with it. Section II outlines the experimental setup, behavioral data collection, as well as acoustic features used to analyze the stimulus set. Section III presents the results of the behavioral experiment along with analyses of different types of salient events. The results also delve into an acoustic analysis of the dataset in hopes of establishing a link between the acoustic structure of the scene stimuli and the perceived salience of events in those scenes. We conclude with a discussion of the relevance of these findings to studies of bottom-up attention and auditory perception of natural soundscapes.

## II. METHODS

### A. Stimuli

A set of twenty recordings of natural scenes was gathered from various sources including the BBC Sound Effects Library,^24^ Youtube,^25^ and the Freesound database.^26^ Scenes were selected with a goal of covering a wide acoustic range. This range included having dense and sparse scenes, smooth scenes and ones with sharp transitions, homogeneous scenes versus ones that change over time, and so forth. A variety of sound categories was included, such as speech, music, machine, and environmental sounds. All recordings were carefully selected to maintain a subjectively good audio quality. An overview of all scenes included in this dataset is given in Table I.

**TABLE I.** List of the twenty audio recordings of natural scenes used in this study and suggested for use in future studies. Scenes selected to cover a wide range of acoustic environments. Scenes were labeled subjectively as sparse or dense in regards to the density of acoustic objects within.

#	Dur	Sparse	Control	Description	Source
1	2:00	No	No	Nature, Birds	Youtube.com/watch?v=U2QQUoYDzAo
2	2:15	Yes	No	Sporting Event	BBC:Humans/Crowds/CrowdsExterior.BC.ECD48c
3	2:00	No	No	Cafeteria	BBC:Ambience/Cafes/CafesCoffees.BBC.ECD40b
4	2:02	Yes	No	Battle, Guns	Youtube.com/watch?v=TVkqVIQ-8ZM
5	1:21	Yes	No	Airplane, Baby	Soundsnap.com/node/58458
6	1:13	No	No	Fair	Freesoundeffects.com/track/carnival-street-party—42950/
7	1:59	No	No	Classical Music	Youtube.com/watch?v=pKOpdt9PYXU
8	2:01	Yes	No	Bowling Alley	BBC:Ambience/America/America24t
9	2:00	No	No	Egypt Protests	Youtube.com/watch?v=H0ADRvi0FW4
10	2:08	No	No	Store Counter	BBC:Ambience/America/America24k
11	1:57	No	No	Drum Line	Youtube.com/watch?v=c4S4MMvDrHg
12	1:59	No	No	Blacksmith	Youtube.com/watch?v=Ka7b-cicOpw
13	2:13	No	No	Orchestra Tuning	Youtube.com/watch?v=6lwY2Dz15VY
14	2:07	No	No	Japanese Game Show	Youtube.com/watch?v=I5PS8zHBbxg
15	2:22	No	No	Dog Park	Freesound.org/people/conleec/sounds/175917/
16	2:14	No	No	Casino	Freesound.org/people/al_barbosa/sounds/149341/
17	2:00	No	Yes	Piano Concerto	Youtube.com/watch?v=ePFBZjVwfQI
18	1:22	Yes	Yes	Car Chase	BBC:Trans/Land/Vehicles/Motocycles/MotorCycleRacing17c
19	1:59	Yes	Yes	Maternity Ward	BBC:Emergency/Hospitals/MaternityWard
20	1:19	No	Yes	Sailing Barge	BBC:Trans/Water/Ships/ShipsSailing.BBC.ECD22b

Each scene had a duration of approximately two minutes (average of 116 ± 20 s). All wave files were downsampled to 22 050 Hz with a bit rate of 352 kbps. One of the scenes (scene number 13) was originally recorded in stereo, from which only the left channel was used. Scenes were normalized based on the root mean square (RMS) energy of the loudest 1% of each wave file. Alternative normalizations (such as RMS energy across the entire waveform or peak normalization) were deemed inappropriate for regulating the scene amplitudes because they made the sparse scenes too loud and the dense scenes too quiet or the reverse.

### B. Behavioral data collection

Fifty healthy volunteers (ages 18–34, 16 males) with no reported history of hearing problems participated in this study. Subjects were asked if they had any musical training, for example, if they had played an instrument, and if so, how many years of training. Subjects were compensated for their participation, and all experimental procedures were approved by the Johns Hopkins University Homewood Institutional Review Board (IRB).

Behavioral data were recorded using Experiment Builder (SR Research Ltd., Oakville, ON, Canada), a software package designed to interface with the EyeLink 1000 eye tracking camera. Subjects were seated with their chins and foreheads resting on a headrest. Subjects were instructed to maintain fixation on a cross in the center of the screen while they performed the listening task. The eye tracker primarily recorded each participant's pupil size over the course of the experiment. It was calibrated at the beginning of the experiment, with a sequence of five fixation points. Dichotic audio stimuli were presented over Sennheiser HD595 headphones inside a sound proof booth. Sounds were presented at comfortable listening levels, though subjects were able to notify the experimenter if any adjustments were needed. Pupil size and mouse movements were both initially recorded at a sampling frequency of 1 kHz. Mouse movements were later downsampled by a factor of 64. Data were analyzed using matlab software (Mathworks, Natick, MA, USA).

**FIG. 1.**
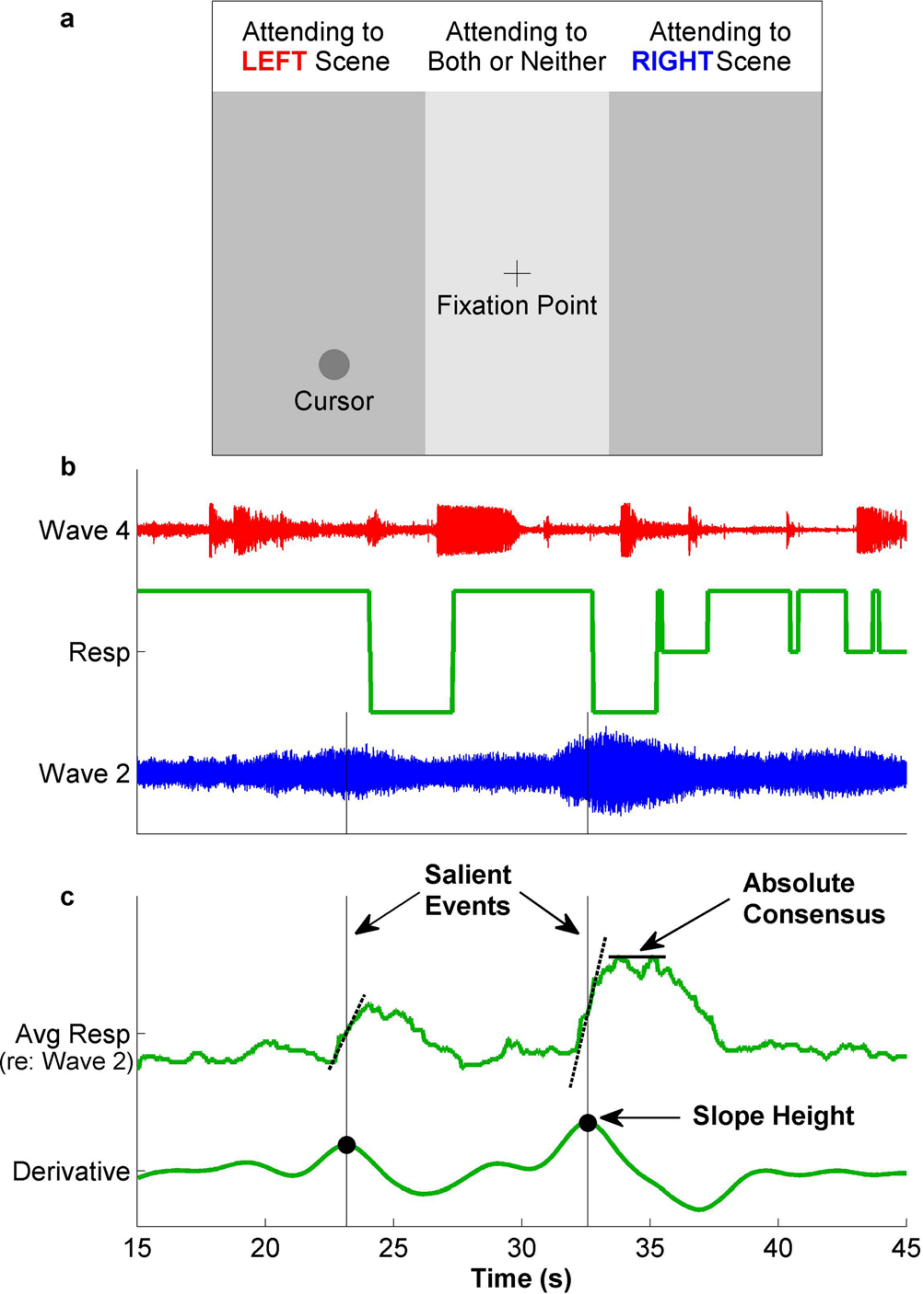
(Color online) (a) Presentation screen for the behavioral task. Subjects were asked to listen to one natural scene in each ear, and to move the cursor to the side corresponding to the scene that they were focusing on at any given time. (Text on top was not part of the presentation screen.) (b) Example waveforms of two stimuli presented in a given trial along with behavioral response of one subject shown in the center. (c) The average response across subjects and competing scenes is shown for waveform 2. Salience is denoted as the percentage of subjects attending to that scene at any given time. Peaks in the height of the slope of the average salience are marked as salient events.

Behavioral responses were collected using a dichotic listening paradigm. Subjects were presented with two different scenes simultaneously, one in each ear. Subjects were instructed to indicate continuously which of the two scenes they were focusing on by moving a mouse to the right or the left of the screen. Subjects were not instructed to listen for any events in particular, but simply to indicate which scene (if any or both) they were focusing on at any given time. A central portion of the screen was reserved for when a subject was either not focusing on either scene or attending to both scenes simultaneously. Subjects were not instructed to differentiate between locations within each region. As such, participants were limited to three discrete responses at any given point in time: left, right, or center. Subjects were also instructed to fixate their gaze on the cross in the center of the screen, in order to avoid effects of eye movements on pupil size. A diagram of the screen is shown in Fig. 1(a).

Before data collection began, subjects were presented with a brief, fifteen second example stimulus, consisting of a pair of natural scenes distinct from the twenty used in the main experiment. The same example was played a second time with a corresponding cursor recording, to serve as one possibility of how they could respond.

Two pairs from the set of twenty scenes used in this study were chosen as “control” scenes, and were always presented with the same opposing wave file. Thus, each of the four control scenes was heard exactly once by each subject (50 trials across subjects). The remaining sixteen scenes were presented in such a way that, across subjects, each scene was paired with each other scene roughly an equal number of times. Each individual subject heard each of these scenes at most twice (total of 62 or 63 trials per scene across 50 subjects), with an average of six scenes presented a second time to each subject. A trial ended when either of the two competing scenes concluded, with the longer scene cut short to that time. There was a fixed rest period of five seconds between trials, after which subjects could initiate the next trial at their leisure.

In parallel, we collected data from 14 listeners in a passive listening mode. In these passive sessions, no active behavioral responses were collected and subjects simply listened to the exact stimuli as described earlier. Only pupil size was collected using the eye tracker.

### C. Analysis of behavioral data

Individual behavioral responses were averaged across subjects for each scene by assigning a value of +1 when a participant attended to the scene in question, −1 when a participant attended to the competing scene, and 0 otherwise [Fig. 1(b)]. Choosing a different range to distinguish the three behavioral states (instead of {+1,0,−1}) resulted in qualitatively similar results. The mean behavioral response across all subjects will henceforth be referred to as the *average salience* for each scene (since it averages across *all* competing scenes). The derivative of this average salience curve was computed and smoothed with three repetitions of an equally weighted moving average with a window length of 1.5 s.^27^ This derivative curve captured the slope of the average salience and was used to define notable moments in each scene. Peaks in the derivative curve were marked as onsets of potential events [Fig. 1(c)]. A peak was defined as any point larger than neighboring values (within 0.064 s). The smoothing operation performed on the average salience curve prior to peak picking eliminated small fluctuations from being selected. The smoothing and selection parameters were selected heuristically and agreed with intuitive inspection of behavioral responses and location of peaks based on eye inspection.

A total of 468 peaks were identified across the twenty scenes. These peaks were ranked based on a linear combination of two metrics: the height of the derivative curve (*slope height*), which reflects the degree of temporal agreement between subjects; and the maximum height of the average salience curve within four seconds following the event onset (*absolute consensus*) which reflects the percentage of subjects whose attention was drawn by an event irrespective of timing. The four second range was chosen in order to avoid small false peaks close to the event, as well as peaks far away that may be associated with later events. The linear combination of these two metrics (*behavioral salience*) was used in order to avoid the dominance of either particularly salient or particularly sparse scenes. The absolute consensus was scaled by the 75th percentile of slope of salience. The most salient 50% of these peaks (234 events) were taken as the set of *salient events* used in the analysis reported in this article, except where indicated.

Events were manually classified into specific categories after their extraction. An experimenter listened to several seconds of the underlying scene before and after each event in order to make the classification. The categories of events were speech, music, other human vocalizations (e.g., laughter), animal sounds, sounds created by a device or machinery, and sounds created by an object tapping or striking another object. Each event was individually categorized into one of these six types.

### D. Acoustic analysis of stimuli

The acoustic waveform of each scene was analyzed to extract a total of nine features along time and frequency, many of which were derived from the spectrogram of each scene. The time-domain signal *s*(*t*) for each scene was processed using the nsl matlab toolbox^28^ to obtain a time-frequency spectrogram *y*(*t*, *x*). The model maps the time waveform through a bank of log-spaced asymmetrical cochlear filters (spanning 255 Hz to 10.3 KHz) followed by lateral inhibition across frequency channels and short-term temporal integration of 8 ms.

(1) The centroid of the spectral profile in each time frame was extracted as a *brightness* measure,^29^ defined as
  $$BR(t)=\frac{\sum_{x}x*y{\left(t,x\right)}^{2}}{\sum_{x}y{\left(t,x\right)}^{2}}.$$
(2) The weighted distance from the spectral centroid was extracted as a measure of *bandwidth*,^30^ defined as
  $$BW(t)=\frac{\sum_{x}|x-BR(t)|*y(t,x)}{\sum_{x}y(t,x)}.$$
(3) *Spectral flatness* was calculated as the geometric mean of the spectrum divided by the arithmetic mean,^31^ also at each time frame,
  $$FL(t)=\frac{{\left(\prod_{x=0}^{N-1}y(t,x)\right)}^{1/N}}{\sum_{x=0}^{N-1}y(t,x)/N}.$$
(4) *Spectral irregularity* was extracted as a measure of the difference in strength between adjacent frequency channels,^32^ defined as
  $$IR(t)=\frac{\sum_{x}{\left(y(t,x+1)-y(t,x)\right)}^{2}}{\sum_{x}y{\left(t,x\right)}^{2}}.$$
(5) *Pitch* (fundamental frequency) was derived according to the optimum processor method by Goldstein.^33^ Briefly, the procedure operates on the spectral profile at each time slice $y(t_{0},x)$ and compares it to a set of pitch templates, from which the best matching template is chosen. The pitch frequency (*F*_0_) is then estimated using a maximum likelihood method to fit the selected template.^34^
(6) *Harmonicity* is a measure of the degree of matching between the spectral slice $y(t_{0},x)$ and the best matched pitch template.
(7) *Temporal modulations* were extracted using the nsl toolbox. These features capture the temporal variations along each frequency channel over a range of dynamics varying from 2 to 32 Hz. See Ref. 28 for further details.
(8) *Spectral modulations* were extracted using the nsl toolbox. These modulations capture the spread of energy in the spectrogram along the logarithmic frequency axis; as analyzed over a bank of log-spaced spectral filters ranging between 0.25 and 8 cycles/octave. See Ref. 28 for further details. For both temporal and spectral modulations, the centroid of each of these measures was taken as a summary value at each time frame.
(9) *Loudness* in bark frequency bands was extracted using the algorithm from the Acoustics Research Centre at the University of Salford.^35^ The process starts by filtering the sound using a bank of 28 log-spaced filters ranging from 250 Hz to 12.5 kHz, each filter having a one-third octave bandwidth to mirror the critical bands of the auditory periphery. The outputs of each filter are scaled to match human perception at each frequency range. Loudness was averaged across frequency bands, providing a specific representation of the temporal envelope of each waveform.

The collection of all nine features resulted in a 9 × *T* representation. Finally, features were z-score normalized to facilitate combining information across features.

### E. Event analysis

Event prediction was conducted on the acoustic features using a similar method to that for extracting behavioral events. This procedure serves as the basis for the simple model to be evaluated in Sec. III. The derivative of each feature was calculated, and then smoothed using the same moving window average procedure used in the behavioral analysis. Peaks in this smoothed derivative were taken as predicted events for each specific feature. The height of this peak was retained as a measure of the strength of this predicted event, allowing for thresholding of events based on this ranking later in the analysis. Thus, a series of events was predicted for each of the nine acoustic features included in this study.

In order to assess the correspondence between events predicted by the acoustic features and behavioral events, the scenes were analyzed over overlapping bins of length *T_B_*. Results reported here are for two-second time windows with a time step of half a second, but qualitatively similar results were observed for other reasonable values of *T_B_*. Each bin containing both a behavioral event and a predicted event was determined to be a “hit”; each bin with only a predicted event was determined to be a “false alarm.” A Receiver operating characteristic or ROC curve could then be generated by varying a threshold applied to the predicted events.

This analysis was performed using events predicted from individual features (e.g., loudness only) or across combinations of features. The cross-feature analysis was achieved in two ways: (a) A simple method was used to flag a predicted event if *any* feature indicated an event within a given bin. (b) Cross-feature integration was performed using Linear Discriminant Analysis (LDA), using ten-fold cross validation.^36^ Each time bin was assigned the value of the strongest event predicted within that bin for each feature, or zero if no such event was present. The acoustic features, and thus the strength of the corresponding predicted events, were z-score normalized. Events from both increases and decreases in each acoustic feature were included in the LDA analysis. Each bin was assigned a label 1 if a behavioral event was present, and 0 if not. Training was done across scenes, with 10% of the data reserved for testing, using contiguous data as much as possible. The process was repeated nine times using a distinct 10% of the data for testing each time. An ROC curve was again generated by varying a threshold on the predicted events.

An ROC curve representing the inter-observer agreement was also generated as a rough measure of the theoretical limit of predictability. This curve was generated by again dividing the scenes into overlapping bins, with the value of each bin corresponding to the percentage of subjects who began attending to the scene in the corresponding time frame. This time series was then thresholded at varying levels and compared to the same set of behavioral events in order to plot the ROC.

### F. Pupil response analysis

Pupil data, acquired using the EyeLink 1000 eye tracking camera (SR Research, Ltd., Oakville, ON, Canada) were sampled at 1000 Hz and normalized as follows: all data were z-score normalized, then averaged across all subjects and all scenes to reach one mean trend line. A power law function was fit to this trend curve starting from 3 until 100 s. This region avoids the initial rising time of the pupil response and the higher amount of noise near the end due to a lower number of scenes with that duration. The trend line was then subtracted from the normalized pupil size recordings from each trial. In order to examine the relationship between pupil responses and the acoustic stimuli or subjects' behavioral responses, increases in pupil size were identified using a similar procedure as the extraction of salient events. The derivative of the pupil size measure was first computed. This derivative was smoothed using the same moving window average procedure used in the behavioral analysis. Peaks in this smoothed derivative were marked as pupil dilations.

## III. RESULTS

### A. Behavioral results

A total of 234 behavioral events are recorded over the 20 scenes used in the database. The behavioral measures used in this study give an estimate of both absolute consensus (reflecting overall agreement across subjects) and slope height (indicating temporal agreement across subjects). Both measures can be taken as indicator of *salience strength* of a sound event; and indeed both measure are found to be significantly correlated with each other [$\rho (232)=0.26,p=6.1\times 10^{-5}$], indicating that an event judged as strong with one metric will often be determined to be strong based on the other.

The minimum number of events found in a scene is four (a quiet cafeteria scene, with an average of 1.8 events/min), while the densest scene has sixteen events (an energetic piano solo excerpt, with an average of 12 events/min). Some scenes have very clear and distinct events, as evidenced by the high slopes in the average salience curve, indicating that the subjects agree upon when the event occurred more closely in time. Other scenes have comparatively less clear events. The mean absolute height of consensus for all events is 56%, indicating that on average, a little over half of the subjects attended to the scene within four seconds following one of these salient events (as described in Sec. II).

**FIG. 2.**
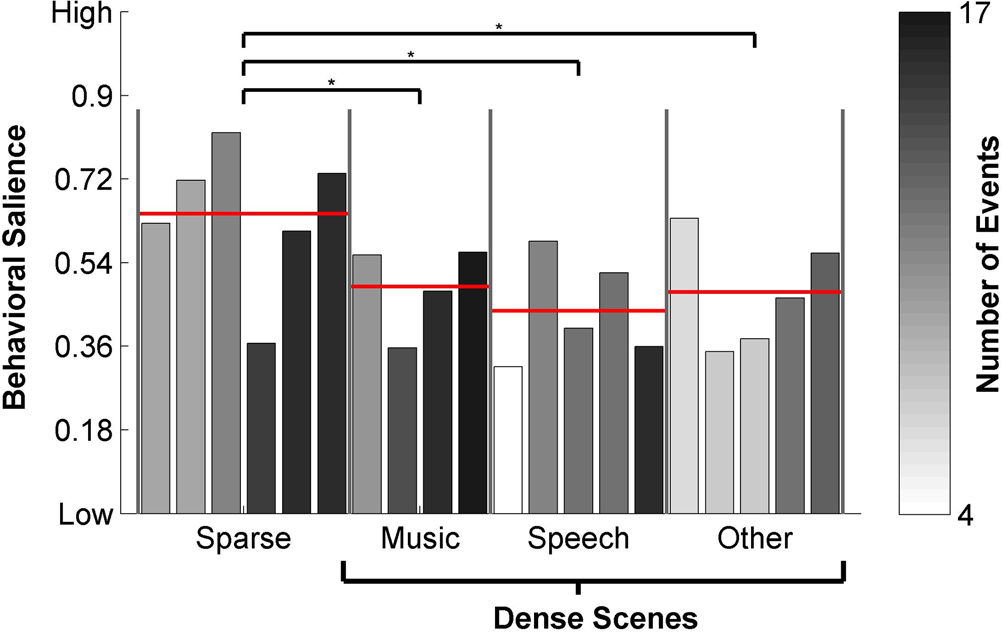
(Color online) Distribution of events across the different natural scenes. Each bar represents one of the twenty scenes in the database. The height of the bar indicates the relative salience strength of the events within that scene. The shading of the bar describes the number of events present within that scene. Scenes are sorted by category, then by number of events within each scene. Sparse scenes contain significantly stronger salient events than the other scene categories, indicated by *.

Figure 2 depicts the number of events and their salience strength for different scenes. Scenes are divided by category: sparse scenes, music scenes, predominantly speech scenes and other. Events in sparse scenes are significantly stronger (as measured by behavioral salience) than events in any of the other three categories (music, $p=2.3\times 10^{-3}$; speech, $p=3.5\times 10^{-5}$; other, $p=3.8\times 10^{-3}$), based on *post hoc* Tukey HSD tests at the 0.05 significance level.

**FIG. 3.**
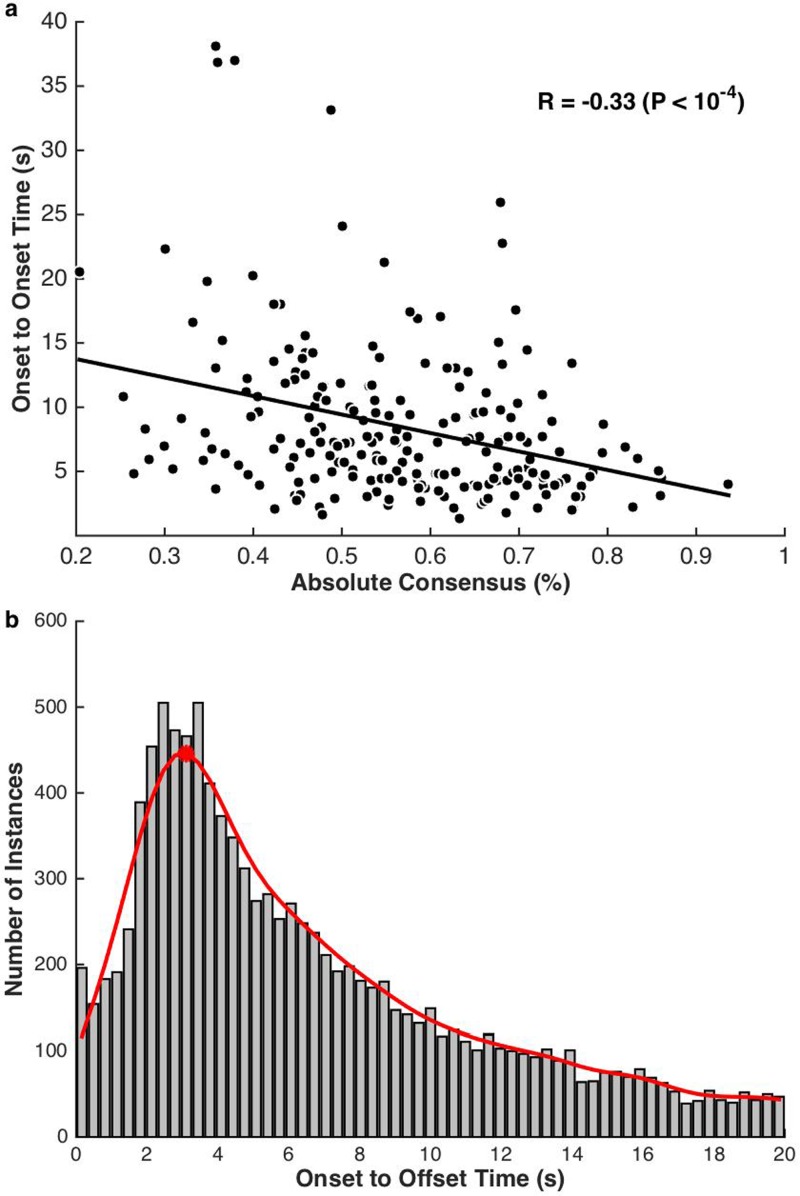
(Color online) (a) The strength of an event relative to the time since the last event. (b) Histogram of individual onset to offset times. These times each correspond to a single mouse movement to and from a scene. A probability density function was fitted using kernel density estimation (a non-parametric method) and peaks at 3.11 s.

Moreover, the time since the last event is inversely correlated with the absolute height of consensus [$\rho (212)=-0.33,\;p=9.04\times 10^{-7}$] [Fig. 3(a)], suggesting that subjects may be sensitized towards a scene after each event. To further assess the timing of salient events, we compute the duration of all shifts in attention towards a scene as the time between when a subject moved the cursor to that scene and when that subject moved the cursor away from it. The distribution of these “onset to offset times” was fit using kernel density estimation and peaks at around 3.11 s [Fig. 3(b)]. This histogram does corroborate the assertion observed in Fig. 3(a) indicating that subjects continue attending to a scene after a salient event 3 s post-onset or more; hence masking any potential behavioral responses within that time window.

### B. Acoustic features

**FIG. 4.**
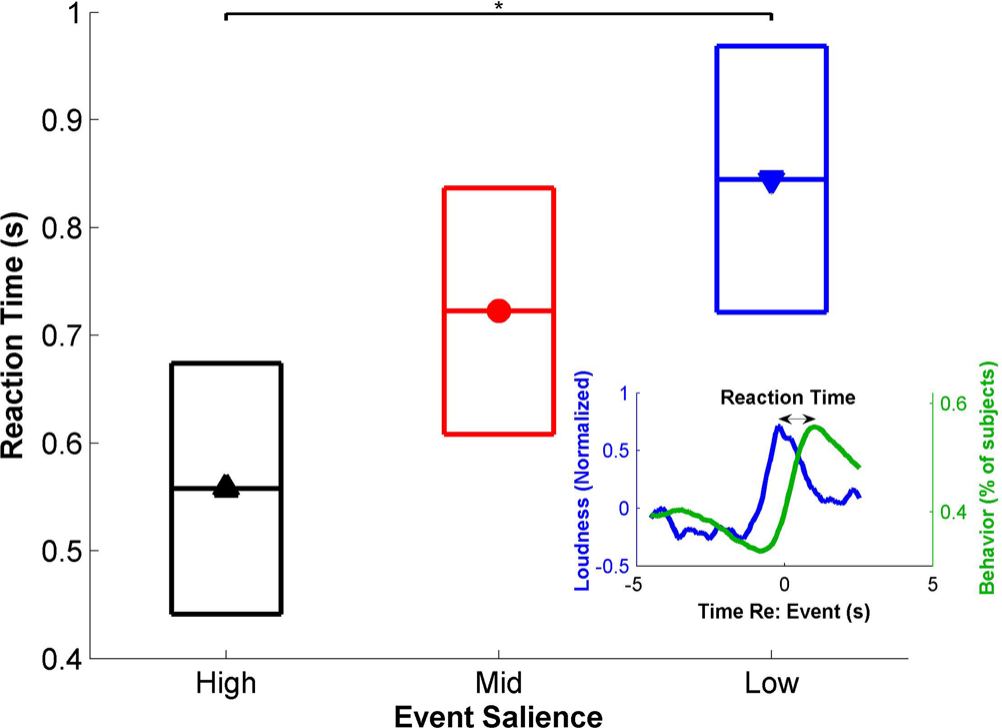
(Color online) Reaction times. The reaction time is defined here as the time between a change in loudness and the change in the behavioral response (inset). Events are ranked by absolute consensus to avoid timing issues. Boxes represent 95% confidence intervals, adjusted for multiple comparisons using a *post hoc* Tukey HSD test.

An acoustic analysis of the scenes sheds light on the attributes of sound events that are deemed salient. On average, subjects tend to respond a little under a second after a change in acoustic properties of the scenes. Using loudness as an example, Fig. 4 (inset) shows the average behavioral response overlaid with the loudness of the scene highlighting the roughly 1 s shift between acoustic change in the scene and the behavioral response. Looking closely at these reaction times, we divide salient events by their strength as quantified by absolute consensus without the slope of salience, to avoid any influence of timing on the rankings. The reaction time is calculated for each behavioral event by finding the time of the maximum slope of loudness in a two second window preceding the behavioral event. Figure 4 shows the confidence intervals (95%) of reaction times for different salience levels, adjusted for multiple comparisons. A *post hoc* Tukey HSD test shows that subjects respond significantly more quickly for high salience events than for low salience events (*p* = 0.047).

**FIG. 5.**
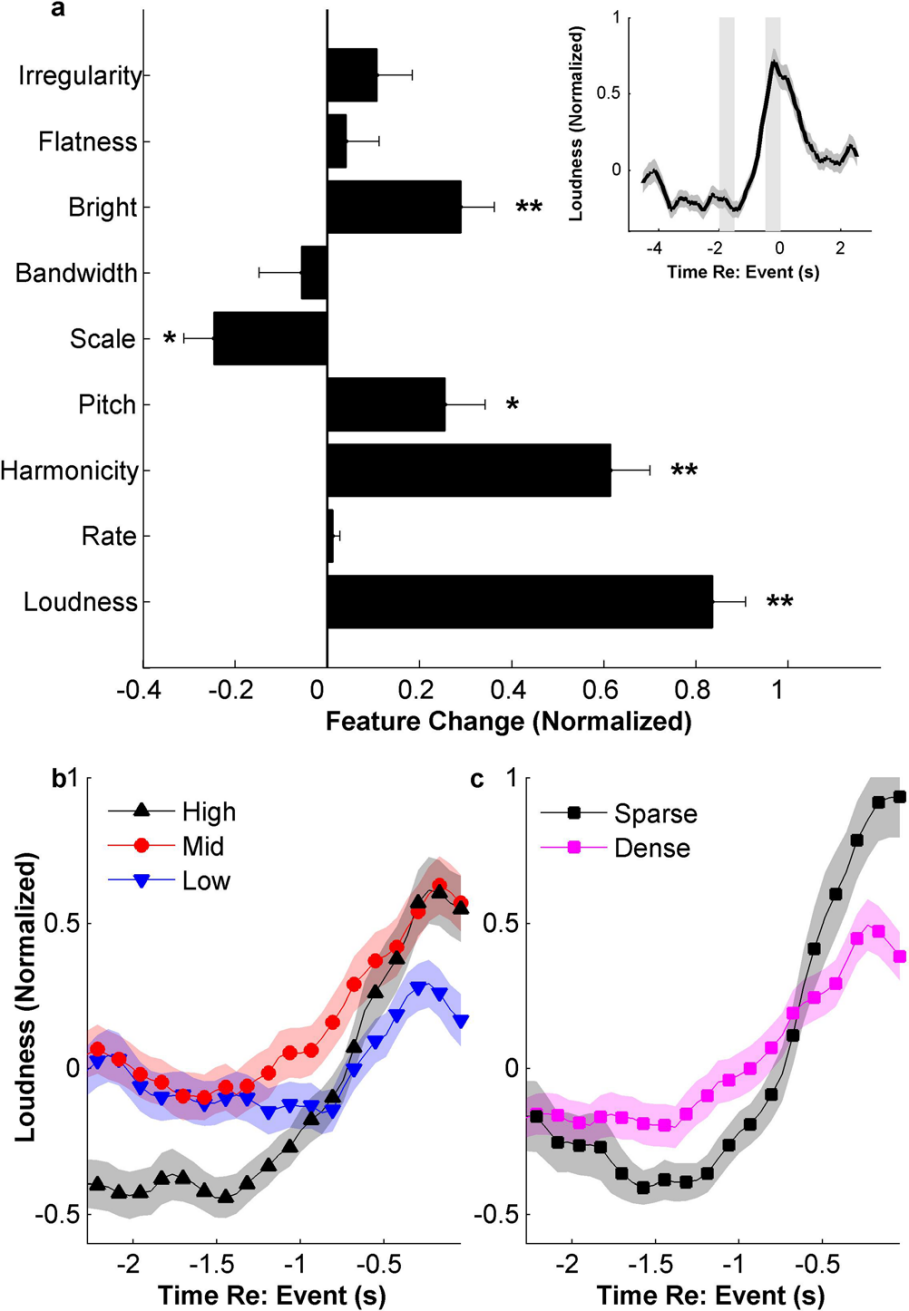
(Color online) (a) Acoustic feature change averaged across events. All features are z-score normalized. Statistically significant increases or decreases are labeled by asterisks, where * indicates *p* < 0.05 and ** p<0.0001. Inset: Average loudness relative to salient events. Vertical bars indicate the time windows used to measure the change in acoustic feature across events. (b) Average loudness around salient events, separated by strength of salience. (c) Average loudness around salient events, separated by scene density. All error bars and shaded areas represent ±1 standard error.

Previous studies have indicated that loudness is indeed a strong predictor of salience.^19^ The current dataset supports this intuitive notion. Figure 5(a) (inset) shows a significant increase in loudness preceding an event, calculated as the average loudness within 0.5 s before an event minus the average loudness between 2.0 and 1.5 s before the event [t(232)=11.6,p≪0.001]. These time ranges relative to the behavioral event are chosen in accordance to the delay of a little under a second between when an acoustic feature changes and when subjects respond (Fig. 4).

In addition to loudness, changes in other acoustic features also correlate with presence of salient events. Figure 5(a) depicts changes in each feature following salient events, normalized for better comparison across features. There are significant increases in brightness [$t(232)=4.0,\;p=9.1\times 10^{-5}$], pitch [t(232)=3.0,p=0.0033], and harmonicity [$t(232)=7.3,p=5.2\times 10^{-12}$], as well as a significant decrease in scale [$t(232)=-3.8,p=1.9\times 10^{-4}$].

In order to extend our analysis beyond just the salient events used in this paper, we examine *all peaks* in the behavioral response (beyond the top 50% retained in most analyses, as outlined in Sec. II). The strength of derivative peaks of these responses correlate with degree of change in acoustic features. Specifically, loudness ($\rho =0.44,\;p=1.0\times 10^{-23}$), harmonicity ($\rho =0.33,\;\;p=2.7\times 10^{-13}$), brightness ($\rho =0.17,\;p=1.95\times 10^{-4}$), and scale (ρ=−0.14,p=0.002) are all statistically significantly correlated with degree of behavioral salience. In other words, the weaker the change in acoustic features, the weaker the behavioral response; hence our choice to focus on the stronger (top 50%) changes since they correlate with better defined acoustic deviations.

### C. Acoustic context

In addition to the absolute value of acoustic features immediately related to a salient event, the acoustic context preceding the event plays an important role in affecting the percept of salience. For instance, an event induced with a given loudness level does not always induce a fixed percept of salience. Instead, the context in which this event exists plays a crucial role, whereby a loud sound is perceived as more salient in a quieter context than in a raucous one. Figure 5(b) shows the average loudness over time around salient events, ranked into three tiers by salience strength. The figure highlights the fact that the events with the highest salience are preceded by a baseline loudness that is lower than average. Specifically, while both high and mid-salience events tend to be equally loud *acoustically*, their perceived salience is different given the loudness of their preceding contexts. Figure 5(c) also shows the average loudness over time for events split by scene density. There is a much larger loudness change for events in sparse scenes.

### D. Event types

**FIG. 6.**
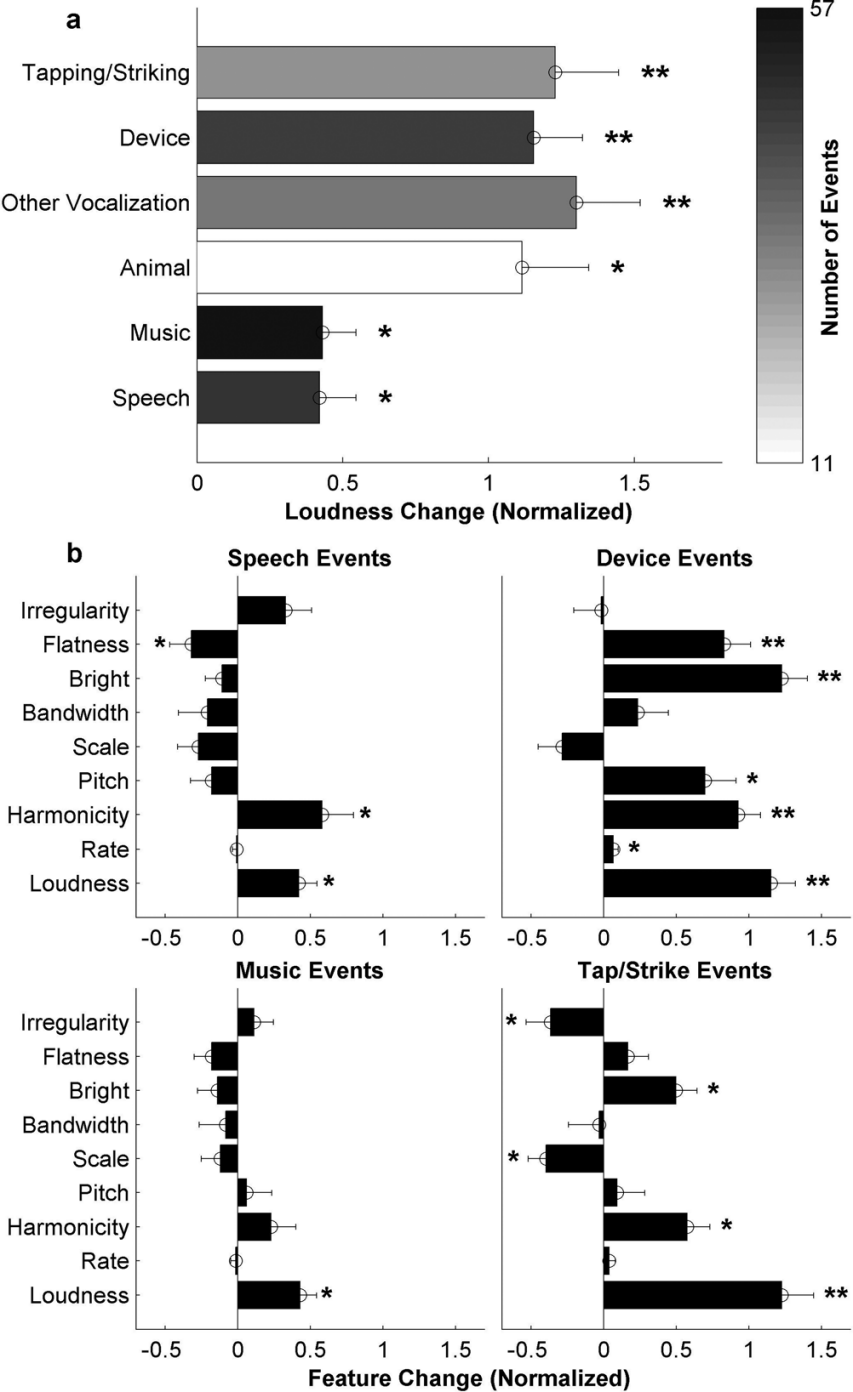
(a) Number of events in each category, and the loudness change associated with each (bottom, * indicates *p* < 0.05, ** indicates *p* < 0.0001). (b) Feature change associated with specific categories of events (* indicates *p* < 0.05, ** indicates *p* < 0.001). All error bars represent ±1 standard error.

Although loudness shows a strong average increase across all events, that increase is not uniform across all types of events, revealing a nonlinear behavior across contexts. In order to better understand the effect of the scene context around the event on its perceived salience, events are manually categorized into different types based on preceding scene immediately prior to the event. Note that many scenes are heterogeneous and include events of different types. The most straight-forward categories are speech, music, and animal sounds. “Other vocalizations” include laughter, wordless cheering, and crying. Of the remaining events, most are either created by some sort of device or machinery (“device”), or associated with objects impacting one another (“tapping/striking”). The number of events in each category are shown in Fig. 6(a). As revealed in this plot, music and speech events do not necessarily exhibit strong changes in loudness and are significantly softer when compared to other event categories [$t(223)=-5.71,\;p=3.7\times 10^{-8}$]. In particular, *post hoc* Tukey HSD tests indicate that speech and music events both show significantly lower loudness increases than any of the top three categories (other vocalization, device, and tap/strike events) at the 0.05 significance level.

In order to further examine the acoustic features associated with events of different types, the histogram of feature changes is broken down into different subcategories [Fig. 6(b)]. This figure is an expansion of Fig. 5(a) that hones in on context-specific effects. The figure highlights different changes in acoustic features that vary depending on context; such as a prominent increase in harmonicity associated with speech events versus a notable change in spectral metrics such as brightness and flatness for more percussive events such as devices [Fig. 6(b), rightmost panels].

### E. Long-term context

**FIG. 7.**
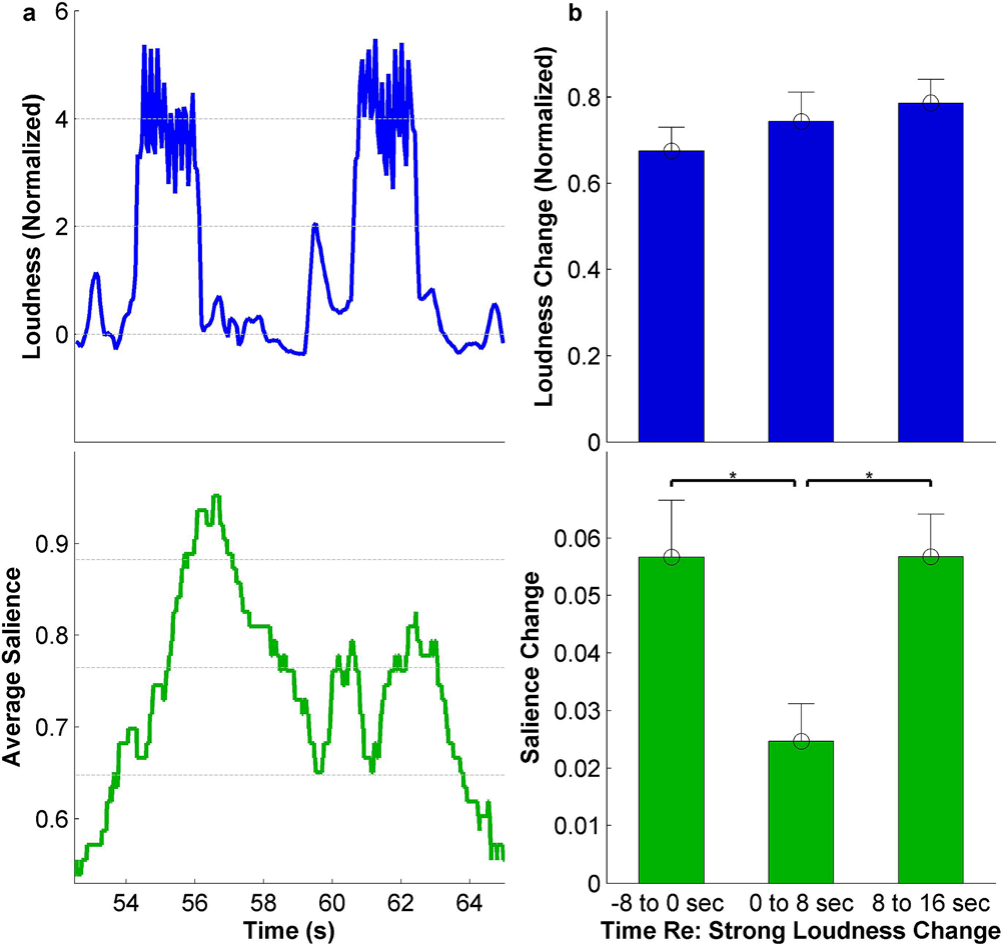
(Color online) (a) An example of a stimulus that produces a time-varying response. A repeated knocking sound elicits a lower response to the second knock. (b) Effects of strong loudness increases on neighboring loudness increases. Statistically significant differences in the response to these acoustic changes are labeled by *.

Although salient events are associated *on average* with changes in acoustic features, there are many instances in which a change in acoustic feature does not result in a behaviorally defined event. Figure 7(a) shows an example of a knocking sound consisting of two consecutive knocks. While both knocks elicit equally loud events [Fig. 7(a), top], the behavioral response (bottom) is markedly reduced to the second knock, presumably because its pop-out factor is not as strong given that it is a repetitive event based on recent history.

In order to evaluate the effects of long-term context more comprehensively, we tally the behavioral responses before and after the strongest loudness changes in our stimuli (top 25% of all loudness changes). On the one hand, we note that loudness changes before ([−8,0] s) and after ([0,8] s and [8,16] s) these strong loudness deviations are not significantly different [Fig. 7(b) top]. On the other hand, loudness increases in a [0,8] s window are found to elicit a significantly lower response than both increases in a eight-second window prior to these events [t(381)=2.68,p=0.0077] and increases in the following eight seconds [t(413)=3.2,p=0.0015] [Fig. 7(b), bottom]. This analysis indicates that acoustic changes over a longer range (up to 8 s) can have a masking effect that can weaken auditory salience of sound events.

### F. Event prediction

Based on the analysis of acoustic features in the scene, we can evaluate how well a purely feature-driven analysis predicts behavioral responses of human listeners. Here, we compare predictions from the features presented in this study using a direct combination method as well as cross-feature combination (see Sec. II). We also contrast predictions from three other models from the literature. The first comparison uses the Kayser *et al.* model^15^ which calculates salience using a center-surround mechanism based on models of visual salience. The second is a model by Kim *et al.*,^19^ which calculates salient periods using a linear salience filter and linear discriminant analysis on loudness. The third is a model by Kaya *et al.*,^18^ which detects deviants in acoustic features using a predictive tracking model using a Kalman filter. While the first two models are implemented as published in their original papers, the Kaya model is used with features expanded to include all the features included in the current study. All three models are trained using cross-validation across scenes. Hit and false alarm rates are calculated using two-second bins (see Sec. II).

**FIG. 8.**
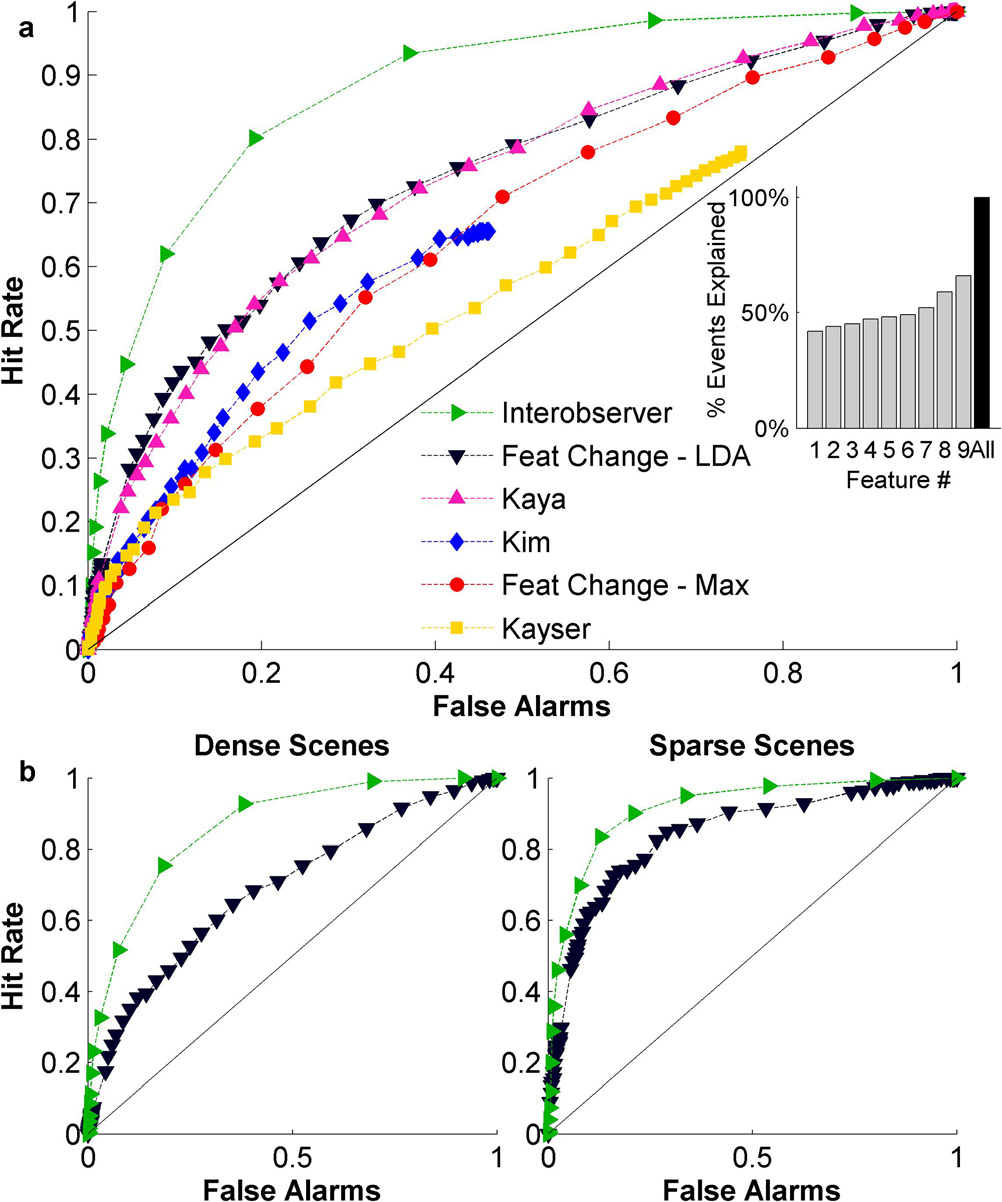
(Color online) (a) Comparison between different methods of predicting salient events. The inset shows the maximum number of events predicted by each individual feature, and by the combination of all features. Features in order are: 1—Flatness, 2—Irregularity, 3—Bandwidth, 4—Pitch, 5—Brightness, 6—Rate, 7—Scale, 8—Harmonicity, 9—Loudness. (b) ROC for dense and sparse scenes separately, using the prediction with the highest performance.

Figure 8 shows a receiver operating characteristic or ROC curve contrasting hit rates and false alarms as we sweep through various thresholds for each model in order to predict behavioral responses of salient events. As is immediately visible in this plot, none of the models is achieving a performance that comes very close to the theoretical limit of predictability. The latter is computed here using a measure of inter-observer agreement (see Sec. II for details). Comparing the different methods against each other, it is clear that a model like Kayser's is limited in its predictive capacity given its architecture as mainly a vision-based model that treats each time-frequency spectrogram as an image upon which center-surround mechanisms are implemented (accuracy of about 0.58, defined as the area under the ROC curve). Given the highly dynamic and heterogeneous nature of the dataset used here, the corresponding spectrograms tend to be rather busy ‘images’ where salient events are often not clearly discernible.

In contrast, the model by Kim *et al.* was designed to map the signal onto a discriminable space maximizing separation between salient and non-salient events using a linear classifier learned from the data. Despite its training on the current dataset, the model is still limited in its ability to predict the behavioral responses of listeners, with an accuracy of about 0.65. It is worth noting that the Kim *et al.* model was primarily designed to identify periods of high salience, rather than event onsets. Moreover, it was developed for the AMI corpus, a dataset that is very homogeneous and sparse in nature. That is certainly not the case in the current dataset.

The Kaya model performs reasonably well, yielding an accuracy of about 0.71. Unlike the other models, the Kaya model attempts to track changes in the variability of features over time in a predictive fashion. It is worth noting that extending the features of the original Kaya model to the rich set employed in the current study was necessary to improve its performance. Interestingly, a relatively simple model which simply detects changes in acoustic features and integrates across these features in a weighted fashion using linear discriminant analysis [“*Feat Change–LDA*” in Fig. 8(a)] performs equally well as the Kaya model. On average, this model is able to predict human judgments of salience with an accuracy of about 0.74 using a linear integration across features. Of note, a simpler version of the feature change model that treats all features as equally important remains limited in its predictable accuracy (about 0.63). This result further corroborates observations from earlier studies about the important role of interaction across acoustic features in determining salience of auditory events.^18,37^ Also worth noting is that smoothing the behavioral responses helps stabilize the detection of change points in the acoustic features. Employing the LDA-based model with raw acoustic features yields an accuracy of only 0.59.

In order to get a better insight of the contribution of different acoustic features in accurately predicting salient events, Fig. 8(a) (inset) examines the proportion of events explained by certain features alone. The analysis quantifies the maximum hit rate achievable. The figure shows that loudness is the single best feature, as it can explain about 66% of all events alone. The next best feature, harmonicity, can explain 59% of events alone. Combining all features together (rightmost bar) is able to explain all events (100%), even though it also comes with false alarms as shown in the ROC curve [Fig. 8(a)]. Qualitatively similar results about relative contribution of features are obtained when generating an ROC curve using each of these features individually. Moreover, the relative contribution of acoustic features to the prediction of events is consistent with an analysis of LDA classifier weights which also show that loudness is the strongest indicator of event salience, followed by harmonicity and spectral scale.

In order to examine the effect of scene structure on the predictability of its salient events based on acoustics, we look closely at predictions for sparse vs less sparse scenes. As noted earlier in this paper, sparse scenes tend to give rise to more events. Also, by their very nature, sparse scenes tend to be quieter which makes changes to features such as loudness more prominent, resulting in stronger salience events [as discussed in Fig. 5(b)]. The sparse scenes with well-defined events may be more easily described by loudness alone. This hypothesis is supported by manually separating the twenty scenes used here into sparse and dense scenes, and evaluating the model's performance on each subset. The prediction for dense scenes with more overlapping objects shows more of an improvement with the inclusion of additional features in comparison to the prediction for sparse scenes. Using maximum hit rate as a metric, loudness alone was only able to account for 58% of salient events in dense scenes, while it could explain 82% of events in sparse scenes. In addition, the event prediction for the dense scenes is closer to the inter-observer ROC than that of the sparse scenes.

**FIG. 9.**
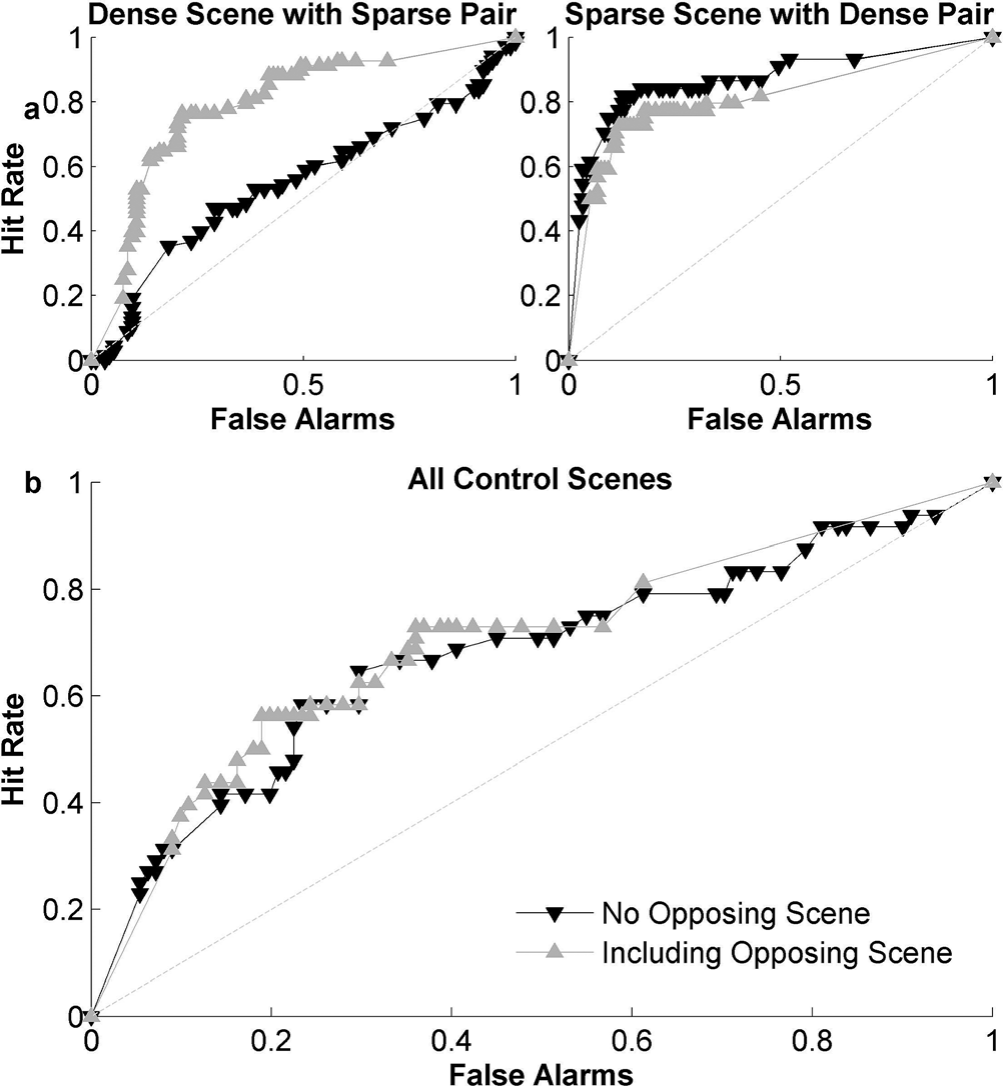
Effects of adding opposing scene information. (a) Examples of prediction performance for two specific scenes, with and without features from the opposing scene. (b) ROC over all control scenes.

For the four “control” scenes, which were always paired with the same competing scene, it is interesting to examine the impact of the opposite scene on salience of a scene of interest. As noted throughout this work, auditory salience of a sound event is as much about that sound event as it is about the surrounding context including a competing scene playing in the opposite ear. We include features of this opposite scene in the LDA classifier. The prediction for one scene shows great benefit [Fig. 9(a)], since it is paired with a very sparse scene containing highly distinct events. However, this effect was not consistent. The prediction of events in a very sparse scene are hindered slightly by including information from the opposing scene [Fig. 9(a)], further reinforcing the idea that event prediction is much more effective on sparse scenes. The other control pair, which involved two more comparable scenes in terms of event strength, are mostly unaffected by including features from the competing scene.

### G. Pupillary response

**FIG. 10.**
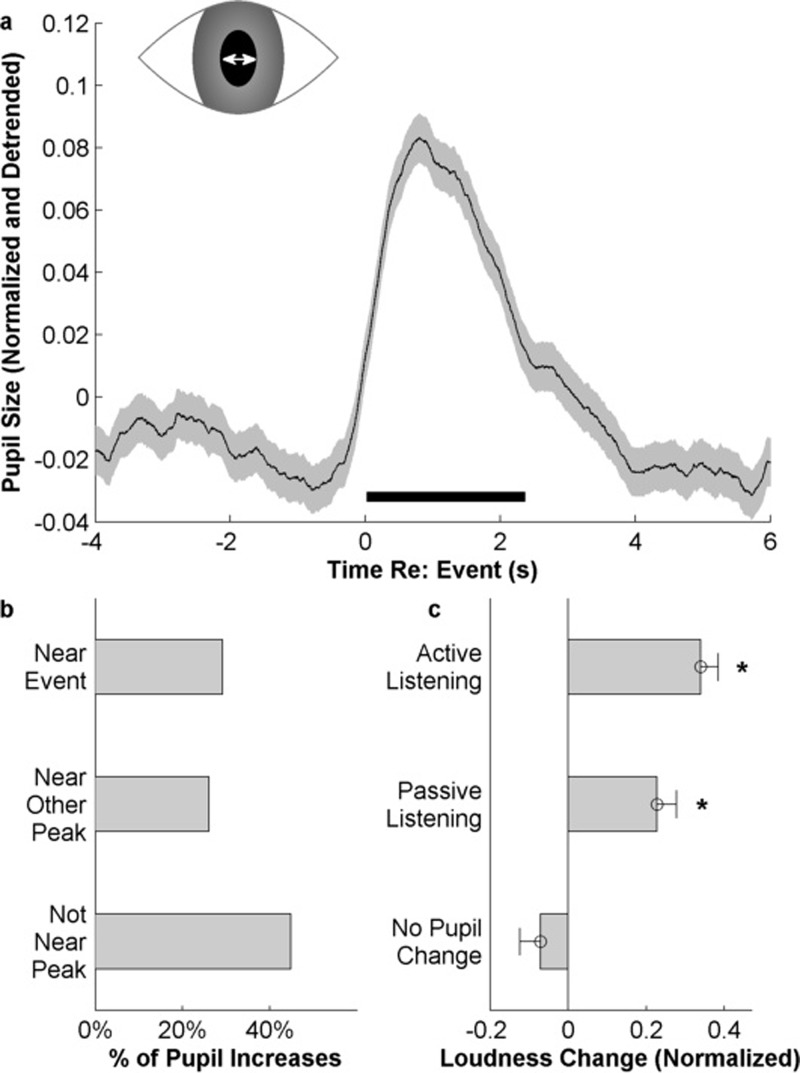
(a) Average pupil size following events, z-score normalized and detrended. The black bar represents the region where the pupil size is significantly greater than zero. (b) Percentage of pupil size increases that are near salient events or other peaks in the slope of salience. (c) Change in loudness associated with increases in pupil size. Error bars represent ±1 standard error.

In addition to recording behavioral responses from listeners, our paradigm monitored their pupil diameter throughout the experiment. The subjects' pupil size shows a significant increase immediately following events defined using these methods [Fig. 10(a)]; with a typical pupil dilation associated with a salient event lasting about three seconds [Fig. 10(a)]. While *all* salient events correlate with pupil dilation, *not all* dilations correlate with salient events. Figure 10(b) shows that about 29% of pupil dilations coincide with salient events (defined as occurring within 1 s of such an event). Another 26% coincide with other peaks in the behavioral responses that we did not label as significant salient events (see Sec. II). Interestingly, 49% of pupil dilations did not coincide with *any* peak in the behavioral response. In contrast, there is a strong correspondence between pupil dilations and changes in acoustic features in the stimuli. An analysis of *all* pupil dilations correlates significantly with increases in acoustic loudness during the behavioral task [$t(548)=7.42,p=4.4\times 10^{-13}$] [Fig. 10(c), top bar]. At the same time, randomly selected points in the pupil responses that do not coincide with any significant pupil variations also *do not* coincide with any significant changes of acoustic loudness [Fig. 10(c), bottom bar]. Finally, in order to rule out any possibility of motor factors during the behavioral task contaminating the pupil response analysis, we replicate the same analysis of pupil changes vs loudness changes during a passive listening session of the same task with no active responses from subjects (N = 14). The results confirm that pupil dilations significantly correlate with increases in acoustic loudness even during passive listening [$t(355)=4.53,\;p=8.2\times 10^{-6}$] [Fig. 10(c), middle bar].

## IV. DISCUSSION

In this study, we present a database of diverse natural acoustic scenes with a goal to facilitate the study and modeling of auditory salience. In addition to the sound files themselves, a set of salient events within each scene has been identified, and the validity of these events have been checked using several methods. Specifically, our analysis confirms the intuition that a change in some or all acoustic features correlates with a percept of salience that attracts a listener's attention. This is particularly true for loud events; but extends to changes in pitch and spectral profile (particularly for speech and music sound events). These effects fit well with contrast theories explored in visual salience.^6^ In the context of auditory events, the concept of contrast is specifically applicable in the temporal dimension, whereby as sounds evolve over time, a change from a low to a high value (or vice versa) of an acoustic attribute may induce a pop-out effect of the sound event at that moment. While earlier studies have emphasized the validity of this theory to dimensions such as loudness,^16,19,22^ our findings emphasize that the salience space is rather multidimensional spanning both spectral and temporal acoustic attributes. Increases in brightness and pitch both suggest that higher frequency sounds tend to be more salient than lower frequency sounds. Naturally, this could be correlated with changes in loudness that are associated with frequency^38^ or more germane to how high frequencies are perceived. The rise in harmonicity suggests that a more strongly pitched sound (e.g., speech, music) tends to be more salient than a sound with less pitch strength. Interestingly, the scale feature (representing a measure of bandwidth) tended in the opposite direction. Events that are more broadband and spectrally spread tended to be more salient than more spectrally narrow events.

The relative contribution of various acoustic features has been previously explored in the literature. Kim *et al.*^19^ reported that they observed no improvement to their model predictions with the incorporation of other features besides sound loudness in Bark frequency bands. However, in the present study, features other than loudness do show a contribution. Loudness is only able to account for around two-thirds of the salient events, with the remaining events explained by one or more of the other eight acoustic features [Fig. 8(a), inset]. In particular, the other features seem helpful in establishing salient events in the context of acoustically dense scenes, in which the difference in loudness between the background and the salient sound is much lower. This finding is supported by Kaya *et al.*^39^ which showed that the contribution to salience from different features varied across sound classes. The Kaya *et al.* study used comparatively more dense acoustic stimuli.

In addition to its multidimensional nature, the space of auditory salience appears to also be context or category-dependent. By using a diverse set of scenes of different genres (e.g., speech, music, etc.), our results indicate that observed contrast across acoustic attributes does not uniformly determine the salience of an auditory event, but rather depends on general context in which this event is present. Notably, speech events appear to be strongly associated with increases in harmonicity. This result aligns with expectations, as speech is a very strongly pitched sound. Similarly, because it is pitched, speech is characterized by a low spectral flatness. As such it is unsurprising that speech events are associated with a decrease in flatness. Also worth noting is that both speech and music events were not associated with any significant or big changes in a number of spectral metrics (including brightness, bandwidth) in contrast with more percussive-type events such as devices and tapping. These latter two contexts induced an expected dominance of loudness [Fig. 6(b), rightmost panels]. However, it is interesting that musical events show the least consistent changes across all features, perhaps indicating that what makes a musical section salient is much more intricate than basic acoustics. Overall, these results suggest that the analysis of salience may need to carefully consider the contextual information of the scene; even though the exact effect of this categorization remains unclear.

Moreover, an interesting observation that emerges from the behavioral judgments of listeners is that auditory salience is driven by acoustic contrast over both short (within a couple of seconds) and long (as much as 8 s) time spans. The multi-scale nature of integration of acoustic information is not surprising given that such processing schemes are common in the auditory system; especially at more central stages including auditory cortex.^40^ However, it does complicate the development of computational models capable of multiplexing information along various time constants; as well as complementing a basic acoustic analysis with more cognitive processes that enable the recognition of contextual information about the scene. This extended analysis over the longer context of a scene will need to delve into memory effects and their role in shaping salience of sound events by reflecting interesting structures in the scene such as presence of repetitive patterns (that may become less conspicuous with repetition) or familiar transitions (as in the case of melodic pieces).

The choice of a diverse set of natural scenes was crucial to painting a fuller picture of this auditory salience space. This follows in the tradition of studies of visual salience; which greatly benefited from an abundance of databases with thousands of images or video with clearly labeled salient regions based on eye-tracking, visual search, identification, and detection measures.^12^ The widespread availability of such databases has greatly facilitated work ranging from visual perception to computer vision. Crucially, the abundance of datasets brought forth a rich diversity of images and videos that challenged existing models and pushed forth advances in our understanding of visual attention and salience. Our goal in the present study is to provide a rich database that takes a step towards achieving the same benefits for the study of auditory salience, providing a level of facilitation and consistency which has previously been unavailable. While the dataset used here represents only a tiny fraction of the diversity of sound in real life, it provides a small benchmark for future models and theories of auditory salience, while stirring the conversation about appropriate selections for development of future datasets.

Scene variety is one of the key qualities of this set of scenes, encouraging the development of models that apply to a wider range of settings. This versatility would be vital to many applications of salience research, such as use in hearing aids and audio surveillance, or as a preliminary filter for scene classification.^41^ Aside from subjective evaluation and comparison of acoustic features, the scene variety in this database is also reflected in the differing behavioral response to each scene. Some scenes had strong, clearly defined events while others had relatively weaker events; some had many events while others had only a few.

As with all studies of salience, the choice of behavioral metric plays a crucial role in defining the perceptual context of a salient event or object. In vision, eye-tracking is a commonly chosen paradigm as eye fixations have been strongly linked to attention^42^ although there is still some influence of top-down attention as seen through task-dependent effects.^43^ Other methods that have been employed include mouse tracking, which is a reasonable approximation to eye tracking while requiring less equipment, even though it is noisier.^44^ Manual object annotation is also common, such as in the LabelMe^45^ and Imagenet^46^ databases. Although manual labeling may be influenced more by top-down information and can consume more time per scene, large amounts of such annotations have been collected using web-based tools.^45^

In the present study, pupil size provides the closest analogy to eye tracking while mouse movements are analogous to mouse tracking. Consistent with previous results in audio and vision studies,^47–49^ our analysis shows that when a salient stimulus is received, pupils naturally dilate to try to absorb as much information as possible. However, using the complex scenes employed in the current study sheds light on a more nuanced relationship between pupil dilations and auditory salience. As the results indicate, pupil dilation correlates strongly with acoustic variations; but not all acoustic variations imply behaviorally salient sound events. In other words, while almost all salient events correlate with an increase in pupil size locally around the event, increases in pupil size do not always coincide with presence of a behaviorally salient event. As such, pupillometry cannot be used as a sole marker of bottom-up salience. It is therefore necessary to engage listeners in a more active scanning of the scenes, since they have to indicate their attentional state in a continuous fashion. Obviously, by engaging listeners in this active behavioral state, this paradigm remains a flawed indicator of salience. To counter that effect, a large number of subjects is sought to balance results against consistency across subjects. Alternative measures based on non-invasive techniques such as EEG and MEG are possible and are being investigated in an ongoing follow-up work.

Despite being an imperfect metric for salience, the behavioral paradigm used in the present study provides a clear account of the onsets of salient events; hence allowing a direct evaluation of the concept of temporal contrast which also champions an analysis of temporal transitions over sound features. Using such a model proves relatively powerful in predicting human judgments of salient events, though limitations in this approach remain due to its local nature in the acoustic analysis, as well as failure to account for the diversity of scenes present. Qualitatively, the results of this study match some of the other trends in the visual salience literature as well. In both, some categories of objects are more difficult than others, in addition to the discrepancy between different scenes. Difficulty in predicting salience in dense acoustic scenes lines up with performance of visual salience models. Borji^50^ recommends focusing on scenes with multiple objects, in part because models perform more poorly for those complex scenes. Similarly, the dense auditory scenes in this study contain events that were much more difficult for any of the existing models to predict. The larger discrepancy between the model predictions and the inter-observer agreement confirms that there is more room for improvement for these scenes. Models of auditory salience need to be designed with these types of stimuli in mind.

In line with the visual literature, our analysis suggests that smoothing of the acoustic features is required to even come close to a good prediction of the acoustic salient events. This is analogous to suggested observations that models that generate blurrier visual maps perform better in the visual domain.^11^ Moreover, models of visual salience incorporating motion in videos have not performed better than static models.^11^ Similarly, some basic attempts at incorporating longer term history in our model of auditory salience were not able to improve predictions. That is not to say, that history plays no role in salience in either field, but that its role may be complex and difficult to model.

Finally, it is worth noting that the lingering issue of the role of top-down attention in the entire paradigm remains unclear despite efforts to minimize its effects. It has been suggested that events with high inter-subject agreement may be associated with true salience, while those with low inter-subject agreement may be associated with top-down attention, because of the variability in what people consciously think is important.^19^ However, since it would be difficult to dissociate events due to top-down attention from simply weaker events resulting from salience, that distinction is not made here. In fact, with the attempts made to minimize effects of top-down attention in this study, it is likely that weaker events identified here are still a result of bottom-up processing.

## References

[1] L. Itti , G. Rees , and J. K. Tsotsos , *Neurobiology of Attention* ( Elsevier, Amsterdam, 2005), pp. 1–203.

[2] J. Driver , “ A selective review of selective attention research from the past century,” British J. Psychol. 92, 53–78 (2001). 10.1348/000712601162103 11256770

[3] F. Baluch and L. Itti , “ Mechanisms of top-down attention,” Trends Neurosci. 34, 210–224 (2011). 10.1016/j.tins.2011.02.003 21439656

[4] S. Treue , “ Visual attention: The where, what, how and why of saliency,” Current Opin. Neurobiol. 13, 428–432 (2003). 10.1016/S0959-4388(03)00105-3 12965289

[5] Marisa Carrasco , “ Visual attention: The past 25 years,” Vision Res. 51, 1484–1525 (2011). 10.1016/j.visres.2011.04.012 21549742 PMC3390154

[6] L. Itti and C. Koch , “ Computational modelling of visual attention,” Nat. Rev. Neurosci. 2, 194–203 (2001). 10.1038/35058500 11256080

[7] J. M. Wolfe and T. S. Horowitz , “ What attributes guide the deployment of visual attention and how do they do it?,” Nat. Rev. Neurosci. 5, 495–501 (2004). 10.1038/nrn1411 15152199

[8] Q. Zhao and C. Koch , “ Learning saliency-based visual attention: A review,” Sign. Process. 93, 1401–1407 (2013). 10.1016/j.sigpro.2012.06.014

[9] A. Borji , M. Cheng , H. Jiang , and J. Li , “ Salient object detection: A benchmark,” IEEE Trans. Image Process. 24, 5706–5722 (2015). 10.1109/TIP.2015.2487833 26452281

[10] D. Parkhurst , K. Law , and E. Niebur , “ Modeling the role of salience in the allocation of overt visual attention,” Vision Res. 42, 107–123 (2002). 10.1016/S0042-6989(01)00250-4 11804636

[11] A. Borji , D. N. Sihite , and L. Itti , “ Quantitative analysis of human-model agreement in visual saliency modeling: A comparative study,” IEEE Trans. Image Process. 22, 55–69 (2013). 10.1109/TIP.2012.2210727 22868572

[12] A. Borji and L. Itti , “ State-of-the-art in visual attention modeling,” IEEE Trans. Pattern Anal. Mach. Intell. 35, 185–207 (2013). 10.1109/TPAMI.2012.89 22487985

[13] S. Frintrop , E. Rome , and H. I. Christensen , “ Computational visual attention systems and their cognitive foundation: A survey,” ACM Trans. Appl. Percept. 7, 1–39 (2010). 10.1145/1658349.1658355

[14] E. M. Kaya and M. Elhilali , “ Modeling auditory attention: A review,” Philos. Trans. R. Soc. B: Biol. Sci. 372(1714), 1–10 (2017). 10.1098/rstb.2016.0101 PMC5206269 28044012

[15] C. Kayser , C. I. Petkov , M. Lippert , and N. K. Logothetis , “ Mechanisms for allocating auditory attention: An auditory saliency map,” Curr. Biol. 15, 1943–1947 (2005). 10.1016/j.cub.2005.09.040 16271872

[16] T. Tsuchida and G. Cottrell , “ Auditory saliency using natural statistics,” in *Proceedings of the Annual Meeting of the Cognitive Science* (2012).

[17] V. Duangudom and D. V. Anderson , “ Using auditory saliency to understand complex auditory scenes,” in *Proceedings of the 15th European Signal Processing Conference* (2007).

[18] E. M. Kaya and M. Elhilali , “ Investigating bottom-up auditory attention,” Front. Hum. Neurosci. 8, 1–12 (2014). 10.3389/fnhum.2014.00327 24904367 PMC4034154

[19] K. Kim , K. Lin , D. B. Walther , M. A. Hasegawa-Johnson , and T. S. Huang , “ Automatic detection of auditory salience with optimized linear filters derived from human annotation,” Pattern Recogn. Lett. 38, 78–85 (2014). 10.1016/j.patrec.2013.11.010

[20] R. Southwell , A. Baumann , C. Gal , N. Barascud , K. Friston , and M. Chait , “ Is predictability salient? A study of attentional capture by auditory patterns,” Philos. Trans. R. Soc. B: Biol. Sci. 372, 1–11 (2017). 10.1098/rstb.2016.0105 PMC5206273 28044016

[21] F. Vachon , K. Labonté , and J. E. Marsh , “ Attentional capture by deviant sounds: A non-contingent form of auditory distraction?,” J. Exp. Psychol.: Learn. Mem. Cogn. (2016). 10.1037/xlm0000330 27656870

[22] V. Duangudom and D. V. Anderson , “ Identifying salient sounds using dual-task experiments,” in *IEEE Workshop on Applications of Signal Processing to Audio and Acoustics* ( Institute of Electrical and Electronics Engineers, Piscataway, NJ, 2013), pp. 1–4.

[23] O. Kalinli and S. Narayanan , “ A saliency-based auditory attention model with applications to unsupervised prominent syllable detection in speech,” in *INTERSPEECH* (2007), pp. 1941–1944.

[24] “The BBC Sound Effects Library—Original Series,” http://www.sound-ideas.com/bbc.html (Last viewed 07/22/2016).

[25] Youtube, http://youtube.com (Last viewed 05/31/2016).

[26] The Freesound Project, http://www.freesound.org (Last viewed 06/06/2016).

[27] S. W. Smith , *The Scientist and Engineer's Guide to Digital Signal Processing* ( California Technical Publishing, San Diego, 1997), pp. 277–284.

[28] T. Chi , P. Ru , and S. A. Shamma , “ Multiresolution spectrotemporal analysis of complex sounds,” J. Acoust. Soc. Am. 118, 887–906 (2005). 10.1121/1.1945807 16158645

[29] E. Schubert and J. Wolfe , “ Does timbral brightness scale with frequency and spectral centroid?,” Acta Acust. Acust. 92, 820–825 (2006).

[30] S. Esmaili , S. Krishnan , and K. Raahemifar , “ Content based audio classification and retrieval using joint time-frequency analysis,” in *Proceedings of the International Conference on Acoustics, Speech, and Signal Processing* ( Institute of Electrical and Electronics Engineers, Piscataway, NJ, 2004), Vol. 5.

[31] J. D. Johnston , “ Transform coding of audio signals using perceptual noise criteria,” IEEE J. Select. Areas Commun. 6, 314–323 (1988). 10.1109/49.608

[32] S. McAdams , J. W. Beauchamp , and S. Meneguzzi , “ Discrimination of musical instrument sounds resynthesized with simplified spectrotemporal parameters,” J. Acoust. Soc. Am. 105, 882–897 (1999). 10.1121/1.426277 9972573

[33] J. L. Goldstein , “ An optimum processor theory for the central formation of the pitch of complex tones,” J. Acoust. Soc. Am. 54, 1496–1516 (1973). 10.1121/1.1914448 4780803

[34] S. A. Shamma and D. J. Klein , “ The case of the missing pitch templates: How harmonic templates emerge in the early auditory system,” J. Acoust. Soc. Am. 107, 2631–2644 (2000). 10.1121/1.428649 10830385

[35] E. Zwicker , H. Fastl , U. Widmann , K. Kurakata , S. Kuwano , and S. Namba , “ Program for calculating loudness according to din 45631 (iso 532b),” J. Acoust. Soc. Jpn. 12, 39–42 (1991). 10.1250/ast.12.39

[36] A. J. Izenman , *Modern Multivariate Statistical Techniques* ( Springer, New York, 2013), pp. 237–280.

[37] F. Tordini , A. S. Bregman , and J. R. Cooperstock , “ The loud bird doesn't (always) get the worm: Why computational salience also needs brightness and tempo,” in *Proceedings of the 21st International Conference on Auditory Display* (2015).

[38] B. C. J. Moore , *An Introduction to the Psychology of Hearing*, 5th ed. ( Emerald Group, Bingley, 2003), pp. 133–167.

[39] E. Kaya and M. Elhilali , “ Investigating bottom-up auditory attention in the cortex,” poster presented at the *38th ARO Midwinter Research Meeting*, Baltimore, MD (2015).

[40] M. Elhilali , J. B. Fritz , D. J. Klein , J. Z. Simon , and S. A. Shamma , “ Dynamics of precise spike timing in primary auditory cortex,” J. Neurosci. 24, 1159–1172 (2004). 10.1523/JNEUROSCI.3825-03.2004 14762134 PMC6793586

[41] O. Kalinli , S. Sundaram , and S. Narayanan , “ Saliency-driven unstructured acoustic scene classification using latent perceptual indexing,” in *IEEE International Workshop on Multimedia Signal Processing* (2009), pp. 478–483.

[42] R. J. Peters , A. Iyer , L. Itti , and C. Koch , “ Components of bottom-up gaze allocation in natural images,” Vision Res. 45, 2397–2416 (2005). 10.1016/j.visres.2005.03.019 15935435

[43] A. L. Yarbus , *Eye Movements and Vision* ( Plenum, New York, 1967), pp. 171–211.

[44] C. Scheier and S. Egner , “ Visual attention in a mobile robot,” in *Proceedings of the IEEE International Symposium on Industrial Electronics* (1997), Vol. 1, pp. SS48–SS52.

[45] B. C. Russell , A. Torralba , K. P. Murphy , and W. T. Freeman , “ Labelme: A database and web-based tool for image annotation,” Int. J. Comput. Vision 77, 157–173 (2008). 10.1007/s11263-007-0090-8

[46] J. Deng , W. Dong , R. Socher , L. Li , K. Li , and L. Fei-Fei , “ Imagenet: A large-scale hierarchical image database,”in *IEEE Conference on Computer Vision and Pattern Recognition* (2009), pp. 248–255.

[47] E. H. Hess and J. M. Polt , “ Pupil size as related to interest value of visual stimuli,” Science 132, 349–350 (1960). 10.1126/science.132.3423.349 14401489

[48] T. Partala and V. Surakka , “ Pupil size variation as an indication of affective processing,” Int. J. Hum. Comput. Stud. 59, 185–198 (2003). 10.1016/S1071-5819(03)00017-X

[49] H. Liao , M. Yoneya , S. Kidani , M. Kashino , and S. Furukawa , “ Human pupillary dilation response to deviant auditory stimuli: Effects of stimulus properties and voluntary attention,” Front. Neurosci. 10, 1–14 (2016). 10.3389/fnins.2016.00043 26924959 PMC4756168

[50] A. Borji , “ What is a salient object? A dataset and a baseline model for salient object detection,” IEEE Trans. Image Process. 24, 742–756 (2015). 10.1109/TIP.2014.2383320 25532178

